# Postnatal care knowledge and perceptions among women in the Oshana region, Namibia

**DOI:** 10.4102/phcfm.v17i1.4738

**Published:** 2025-09-11

**Authors:** Enos Moyo, Perseverance Moyo, Tafadzwa Dzinamarira, Andrew Ross

**Affiliations:** 1 School of Nursing and Public Health, Faculty of Health Sciences, University of KwaZulu-Natal, Durban, South Africa; 2 Clinical Department, Medical Centre Oshakati, Oshakati, Namibia; 3 School of Health Systems and Public Health, Faculty of Health Sciences, University of Pretoria, Pretoria, South Africa; 4 School of Family Medicine, Faculty of Health Sciences, University of KwaZulu-Natal, Durban, South Africa

**Keywords:** postnatal care knowledge, postnatal care perceptions, associated factors, Oshana region, Namibia

## Abstract

**Background:**

Postnatal care (PNC) knowledge and positive perceptions are crucial for women’s utilisation of PNC services.

**Aim:**

The study aimed to assess the level of PNC knowledge, perceptions of PNC, and determinants of both among women.

**Setting:**

Public healthcare facilities in the Oshana region, Namibia.

**Methods:**

The study followed a quantitative cross-sectional survey design. A self-administered questionnaire was administered to 814 participants selected via systematic random sampling. PNC knowledge and perceptions were used separately as dependent variables. Participants’ characteristics were used as independent variables. Chi-square tests and binomial and multinomial logistic regression were used to analyse associations between PNC knowledge or perceptions and participants’ characteristics.

**Results:**

Among the participants, 55.6% (*n* = 434) demonstrated good PNC knowledge, while 27.3% (*n* = 213) had positive PNC perceptions. Participants who had no formal education, were unemployed, and did not utilise PNC services had a lower likelihood of having good PNC knowledge; adjusted odds ratio (AOR) = 0.33, 95% confidence interval (CI), 0.21–0.53, crude odds ratio (COR) = 0.68, 95% CI, 0.49–0.92; and AOR = 0.72, 95% CI, 0.52–0.98. Similarly, women who did not attend antenatal care had a lower likelihood of having positive PNC perceptions; COR = 0.56, 95% CI, 0.33–0.96.

**Conclusion:**

There is a need for multipronged interventions to improve PNC knowledge and perceptions among women in the Oshana region.

**Contribution:**

This study identified context-specific factors that influence women’s PNC knowledge and perceptions.

## Introduction

Sub-Saharan Africa (SSA) experiences a significant share of maternal and newborn mortality, representing 70% of global maternal deaths in 2020.^1^ Approximately 60% of maternal deaths occur during the postpartum period worldwide.^2^ The global neonatal mortality rate (NMR) decreased from 37 deaths per 1000 live births in 1990 to 17 deaths per 1000 live births in 2020. However, SSA continues to experience a high burden, reporting 27 neonatal deaths per 1000 live births in 2020, in contrast to fewer than five in Europe.^3^ Approximately one-third of global child fatalities take place during the neonatal period, which is defined as the initial 4 weeks of life.^2^ Seventy-five per cent of neonatal deaths occur within the first week post-birth.^2^ Postnatal care (PNC) services offer a critical intervention to reduce these grim statistics. Postnatal care includes several care components that evaluate the health of both newborns and mothers and educate postpartum women on self-care and newborn care techniques.^4^ High-quality PNC guarantees that all infant immunisations are received,^5^ safe contraception is accessible to women,^6^ and HIV-positive women are assisted in taking steps to avoid HIV transmission to their child.^7^ However, PNC utilisation in SSA remains low, with only an estimated 52.5% of postpartum women receiving these vital services in 2018.^8^ Failure to utilise PNC services contributes to high maternal and neonatal morbidity and mortality.^9^ Studies have identified a lack of PNC knowledge and negative perceptions of PNC services by women as barriers to PNC utilisation.^9,10^

Postnatal care knowledge empowers women to make informed decisions about utilising postpartum critical services.^11^ Several factors at the individual and healthcare system levels influence women’s PNC knowledge in SSA. Individual level factors include a woman’s age,^2,12^ marital status,^2,13^ parity,^14,15^ employment status^9,16^ and educational attainment.^17,18,19^ Studies consistently show higher PNC knowledge among urban residents,^20,21,22^ married women^2,13^ and women with higher educational attainment.^23,24,25^ Antenatal care (ANC) attendance,^15,17,26,27^ place of delivery, distance to a healthcare facility^9,28^ and prior PNC experience^14,16,20,24^ are a few health system factors influencing PNC knowledge. The perceptions of PNC among women are also influenced by various factors such as age, place of residence, proximity to a healthcare institution, attendance at ANC and knowledge of PNC services.^29^ Women’s prior experiences with maternal healthcare are another factor. Respectful and woman-centred care experiences are linked to positive PNC perceptions.^10^

Postnatal care services can be accessed at Namibia’s public hospitals, health centres, clinics and private healthcare facilities. Namibians can access PNC services free of charge at public healthcare facilities. However, women can still incur costs, such as transport and food while accessing the services, since some stay far from healthcare facilities. Despite these interventions, there is inadequate knowledge of the determinants of PNC knowledge and perceptions in the Oshana region. This study aimed to assess the level of knowledge and perceptions regarding PNC as well as the determinants influencing these factors among women in the Oshana region of Namibia. The findings aim to guide policymakers in developing strategies to improve PNC knowledge and perceptions. Ultimately, this could increase PNC utilisation and potentially reduce the region’s maternal and neonatal morbidity and mortality.

## Research methods and design

### Study setting and period

The research was carried out from 01 October 2023 to 30 January 2024, across all 18 public healthcare facilities in the Oshana region. Approximately 175 000 individuals reside in the Oshana region.^30^ Women make up about 55% of the population, and roughly 55% of the population in the region resides in rural areas.^30^ The region’s public healthcare system includes one hospital, 12 primary health care clinics and five health centres.^31^

### Study design

We have utilised a quantitative cross-sectional survey study design. This design was used because it is suitable for determining associations between variables at a particular moment in time.

### Study population

All women of reproductive age in Namibia’s Oshana region made up the study population.

### Sample size

The size of the sample was calculated using Epi Info version 7.2.4.0. An estimated 8000 women met the study’s inclusion criteria.^30^ The PNC utilisation rate of 52.5% for SSA, reported in 2018,^8^ was employed to calculate the sample size, as determining PNC utilisation was the study’s primary goal. The sample size calculation was performed using a margin of error of 5%, a 95% confidence interval (CI) and an additional contingency of 10%. The study utilises a multistage sampling procedure, resulting in a design effect of 2. The sample size comprised 814 women.

### Inclusion criteria

Participants included women who had given birth within the 5 years prior to the study and were receiving ANC or women receiving PNC at any of the Oshana region’s 18 public healthcare facilities. This timeframe ensured that participants had relatively recent knowledge of PNC services and perceptions relevant to their most recent childbirth experience.

### Exclusion criteria

This study excludes seriously ill women, those unable to speak, those who did not reside in the Oshana region and those who had training in a healthcare-related profession.

### Sampling and data collection

The study used data from a PNC utilisation study conducted by the authors, whose sampling, questionnaire’s reliability and validity as well as data collection procedures were described in another publication.^32^ Briefly, a systematic random sampling method was used to select the participants. A self-administered questionnaire, available in both English and Oshikwanyama, was used to collect the data. The questionnaire’s reliability and validity were determined during a pilot study with 73 participants.

### Data analysis

Coded data were entered into IBM Statistical Package for Social Sciences (SPSS) version 29 for analysis. Postnatal care knowledge and perceptions were separately used as dependent variables, while participants’ characteristics were used as independent variables. We used frequencies and percentages, two descriptive statistics, to analyse nominal and ordinal data. A percentage was given for each response’s frequency. By adding up each participant’s total scores from the relevant questionnaire sections, the authors statistically quantified the participants’ knowledge and perceptions of PNC. Knowledge was classified into good or poor, while perceptions were classified into positive or negative. A participant was deemed to have good PNC knowledge if their total score was within the range of 216–270; if it was 215 or lower, they were deemed to have poor knowledge. Participants with positive PNC perceptions were identified by having 40–50 total scores, whereas scoring 39 or lower was considered to reflect negative PNC perceptions. Chi-square tests were employed to evaluate the relationships between participants’ characteristics and their perceptions of PNC as well as between participants’ characteristics and their knowledge of PNC. Binomial logistic regression was employed to determine the extent of associations that demonstrated statistical significance in Chi-square tests. The Adjusted Odds Ratios (AOR) were calculated through multinomial logistic regression, utilising characteristics that exhibited statistically significant associations in binomial logistic regression. A *p*-value below 0.05 and a 95% CI were utilised to evaluate the statistical significance of the results.

### Ethical considerations

The Biomedical Research Ethics Committee of the University of KwaZulu-Natal (Protocol reference number: BREC/00005788/2023) and the Ministry of Health and Social Services in Namibia (Ref: 22/4/2/3) both approved this study. The researchers explained to all potential participants that participation was voluntary and that there would be no penalties for failure to participate. The researchers ensured that all the participants were above the age of 18 years. Those who decided to participate in the study provided informed consent before completing the questionnaire.

## Results

### Characteristics of participants

A total of 814 participants were selected to participate in the study. However, only 780 participants returned questionnaires that were completed fully, resulting in a response rate of 95.8%. The majority of the participants (*n* = 546; 70%) were receiving ANC services, *n* = 507; 65% were urban residents, *n* = 614; 78.7% had one to three children, *n* = 734; 94.1%, were single *n* = 473; 60.6% had a healthcare facility delivery for their last child, *n* = 631; 80.9% had a normal vaginal delivery for their last child, *n* = 681; 87.3% had an awareness of available PNC services, *n* = 438; 56.2% did not use PNC services and *n* = 417; 53.5% had poor PNC perceptions. Further information is provided in Table 1.

**TABLE 1:** Frequency distribution of characteristics of participants.

Participants’ characteristics	Frequency	
	Number	Percentage (%)
**Age group (years)**		
18–25	171	21.9
26–30	170	21.8
31–35	128	16.5
36–40	125	16.0
41–45	119	15.3
46–50	66	8.5
**Service being received**		
ANC	546	70.0
PNC	234	30.0
**Residence**		
Urban	507	65.0
Rural	273	35.0
**Participant’s status of employment**		
Employed	560	71.8
Not employed	220	28.2
**Highest level of education**		
No formal education	114	14.6
Primary education	252	32.3
Secondary education	263	33.7
Tertiary education	151	19.4
**Number of children**		
1–3	614	78.7
4–6	118	15.1
> 6	48	6.2
**Marital status**		
Single	473	60.6
Married	152	19.5
Divorced	106	13.6
Widowed	49	6.3
**Where the last child was born**		
Healthcare facility	734	94.1
Home	46	5.9
**Perception of PNC**		
Negative	567	72.7
Positive	213	27.3
**How the last child was delivered**		
Caesarean section	149	19.1
Vaginal delivery	631	80.9
**ANC attendance during last pregnancy**		
Yes	682	87.4
No	98	12.6
**Awareness of PNC services**		
No	681	87.3
Yes	99	12.7
**PNC utilisation after the last pregnancy**		
No	438	56.2
Yes	342	43.8

Source: Moyo E, Moyo P, Dzinamarira T, Murewanhema G, Ross A. Postnatal care utilization in the Oshana region of Namibia: Prevalence, associated Factors, and a decision framework. Int J Afr Nurs Sci. 2024;21:100770. https://doi.org/10.1016/j.ijans.2024.100770

ANC, antenatal care; PNC, postnatal care.

### Postnatal care knowledge among participants

Overall, 434 participants (55.6%) had good PNC knowledge, 95% CI, 52.1–59.1, while 346 (44.4%) had poor PNC knowledge, 95% CI, 40.9–47.9.

### Determinants of postnatal care knowledge among participants

According to the results of Chi-square tests, the associations between PNC knowledge and parity, the highest level of education, employment status, ANC attendance during the last pregnancy and PNC utilisation during the last pregnancy were statistically significant (*p* < 0.05). When using secondary education as the reference group, participants without a formal education had a 67% lower likelihood of having good PNC knowledge, AOR = 0.33, 95% CI, 0.21–0.53. Unemployed participants had a 32% lower chance of having good PNC knowledge compared to the employed, Crude Odds Ratio (COR) = 0.68, 95% CI, 0.49–0.92, but the relationship was not statistically significant in multinomial logistic regression. Participants with more than six children had a 57% lower chance of having good PNC knowledge than those who had one to three children, AOR = 0.43, 95% CI, 0.23–0.80. Participants who did not attend ANC visits or did not utilise PNC services had a 39% and 28% lower likelihood of having good PNC knowledge, AOR = 0.61, 95% CI, 0.39–0.96 and AOR = 0.72, 95% CI, 0.52–0.98, respectively. More details are shown in Table 2.

**TABLE 2:** Associations between postnatal care knowledge and characteristics of participants.

Participants’ characteristics	Crude odds ratios	95% CI	Adjusted^†^ odds ratios	95% CI	Chi-square test *p*-value
**Highest level of education**	**-**	**-**	**-**	**-**	**< 0.010**
No formal education	**0.32**	**0.20–0.50**	**0.33**	**0.21–0.53**	-
Primary education	0.96	0.68–1.37	0.99	0.69–1.42	-
Secondary education	Reference	Reference	Reference	Reference	-
Tertiary education	0.77	0.52–1.16	0.75	0.50–1.14	-
**Participant’s status of employment**	**-**	**-**	**-**	**-**	**0.014**
Employed	Reference	Reference	Reference	Reference	-
Not employed	**0.68**	**0.49–0.92**	0.79	0.56–1.10	-
**Parity**	**-**	**-**	**-**	**-**	**< 0.010**
1–3	Reference	Reference	Reference	Reference	-
4–6	0.81	0.54–1.20	0.96	0.63–1.45	-
> 6	**0.40**	**0.22–0.74**	**0.43**	**0.23–0.80**	-
**ANC attendance during last pregnancy**	**-**	**-**	**-**	**-**	**0.012**
No	0.58	0.38–0.89	0.61	0.39–0.96	-
Yes	Reference	Reference	Reference	Reference	-
**PNC utilisation**	**-**	**-**	**-**	**-**	**< 0.010**
No	**0.62**	**0.46–0.82**	**0.72**	**0.52–0.98**	-
Yes	Reference	Reference	Reference	Reference	-
Perception of PNC	-	-	-	-	0.230
Marital status	-	-	-	-	0.560
Where the last child was born	-	-	-	-	0.160
How the last child was delivered	-	-	-	-	0.840
Service being received	-	-	-	-	0.330
Age group (years)	-	-	-	-	0.110
Residence	-	-	-	-	0.640
Awareness of PNC services	-	-	-	-	0.054

Note: Bold numbers show statistically significant results.

CI, confidence interval; ANC, antenatal care; PNC, postnatal care.

† , Adjusted for highest level of education, employment status, parity, ANC attendance during last pregnancy and PNC utilisation.

### Postnatal care perceptions among participants

Overall, 213 (27.3%) participants had positive PNC perceptions, 95% CI, 24.2–30.4, while 567 (72.7%) had negative PNC perceptions, 95% CI, 69.6–75.8.

### Determinants of postnatal care perceptions among participants

According to Chi-square test results, the associations between PNC perceptions and marital status, mode of delivery of the last child, ANC attendance during the last pregnancy, awareness of PNC services and PNC utilisation during the last pregnancy were statistically significant (*p* < 0.05). The study used single women as the reference group and found that married women were statistically more likely to have positive perceptions of PNC, COR = 1.60, 95% CI, 1.08–2.36. However, the association was not statistically significant in multivariate logistic regression. Women who gave birth to their last child by caesarean section had a statistically significantly higher likelihood of having positive PNC perceptions compared to those who gave birth vaginally, COR = 1.69, 95% CI, 1.16–2.47, but the association did not hold in Adjusted Regression Analysis (ARA). Women who were not aware of PNC services or failed to attend ANC during their last pregnancy had statistically significant lower chances of having positive PNC perceptions, COR = 0.55, 95% CI, 0.32–0.95; and COR = 0.56, 95% CI, 0.33–0.96, but the associations were not statistically significant in multivariate logistic regression. Furthermore, women who did not utilise PNC services during their last pregnancy were less likely to have positive PNC perceptions, AOR = 0.43, 95% CI, 0.30–0.61. Further information has been provided in Table 3.

**TABLE 3:** Associations between postnatal care perceptions and characteristics of participants.

Participants’ characteristics	Crude odds ratios	95% CI	Adjusted^†^ odds ratios	95% CI	Chi-square test *p*-value
**Marital status**	**-**	**-**	**-**	**-**	**0.014**
Single	Reference	Reference	Reference	Reference	-
Married	**1.60**	**1.08–2.36**	1.22	0.81–1.84	-
Divorced	0.96	0.59–1.56	1.03	0.63–1.69	-
Widowed	0.47	0.21–1.07	0.49	0.21–1.14	-
**How last child was delivered**	**-**	**-**	**-**	**-**	**< 0.010**
Caesarean section	**1.69**	**1.16–2.47**	1.10	0.73–1.68	-
Vaginal delivery	Reference	Reference	Reference	Reference	-
**ANC attendance during last pregnancy**	**-**	**-**	**-**	**-**	**0.030**
No	**0.56**	**0.33–0.96**	0.67	0.38–1.16	-
Yes	Reference	Reference	Reference	Reference	-
**Awareness of PNC services**	**-**	**-**	**-**	**-**	**0.030**
Yes	Reference	Reference	Reference	Reference	-
No	**0.55**	**0.32–0.95**	0.64	0.37–1.11	-
**PNC utilisation**	**-**	**-**	**-**	**-**	**< 0.010**
No	**0.37**	**0.27–0.51**	**0.43**	**0.30–0.61**	-
Yes	Reference	Reference	Reference	Reference	-
Place of delivery of last child	-	-	-	-	0.590
Service being attended	-	-	-	-	0.060
Age group (years)	-	-	-	-	0.810
Residence	-	-	-	-	0.640
Highest level of education	-	-	-	-	0.710
Participant’s status of employment	-	-	-	-	0.470
Parity	-	-	-	-	0.350
PNC knowledge	-	-	-	-	0.230

Note: Bold numbers show statistically significant results.

CI, confidence interval; ANC, antenatal care; PNC, postnatal care.

† , Adjusted for marital status, how last child was delivered, ANC attendance during last pregnancy, awareness of PNC services and PNC utilisation.

## Discussion

The study findings indicated that just over half (55.6%) of the participants had good PNC knowledge. This is much higher than the 26.9% that a study from Kenya reported.^9^ This difference might be attributable to educational attainment. This study revealed a higher proportion of participants with secondary education relative to the Kenyan study. Consistent with this, participants without formal education had a lower likelihood of having good PNC knowledge compared to those with secondary education. Similar findings were reported in Ethiopia.^33,34^ Secondary education may expose women to information about PNC during science or life skills classes. Additionally, higher educational attainment might enhance a woman’s ability to comprehend PNC information received from healthcare providers or media sources.

This study also revealed that unemployed women had a lower chance of having good PNC knowledge compared to the employed. The findings align with those of a study conducted in Ethiopia^16^ and another in Kenya.^9^ This disparity could be due to employed women having greater access to maternal healthcare services, where they might receive PNC information during visits. Furthermore, women who failed to attend ANC services during their last pregnancy had a lower likelihood of having good PNC knowledge, mirroring previous studies.^22,27^ This study demonstrates that women who did not utilise PNC services following their last delivery exhibited a lower likelihood of possessing adequate PNC knowledge in comparison to those who utilised these services. These findings concur with those of earlier studies,^21,33^ which had reported similar findings. These findings are unsurprising, as exposure to healthcare professionals during ANC and PNC consultations offers a natural opportunity to learn about these services.

The current study reveals that women with more than six children had a lower likelihood of having good PNC knowledge than those with one to three children. These findings are similar to those of an Ethiopian study.^35^ The fact that women with a greater number of children may find it more challenging to attend maternity healthcare services because they have many household responsibilities – this explanation may help to explain these findings. Another explanation may be that they may not have enough income to cover costs related to attending maternal healthcare services because of household expenses, resulting in them not getting enough information about PNC.

The findings of this study indicated no statistically significant association between maternal age and PNC knowledge. This is at variance with two earlier studies,^2,12^ which indicated that an older maternal age was associated with an improvement in PNC knowledge. The results of this study were unexpected since it would be anticipated that older women would know more about PNC because they had likely visited maternity healthcare services in the past and been informed about PNC.

Nonetheless, the results might be a result of the region’s trend of women beginning families later in life. Another unexpected finding in this study was that the place of delivery was not associated with PNC knowledge. This finding contrasts with the results of other studies,^15,17,20^ which revealed that women who gave birth at healthcare facilities had a higher chance of having good PNC knowledge. It is possible that women in the Oshana region do not receive adequate PNC information at healthcare facilities because, as in other regions of the country,^36^ the healthcare workers may have a high workload, limiting their ability to provide sufficient information on PNC to clients.

The current study reveals that only 27.3% of the participants had positive PNC perceptions. This low proportion of positive PNC perceptions can be attributed to several factors. One factor that acted as a barrier to positive PNC perceptions was being single. Therefore, since most participants were single, this might have contributed to the low proportion of positive PNC perceptions among the participants. Married women had a higher likelihood of having positive PNC perceptions than single women. This finding may be explained by the possibility that women who have partners may also receive PNC information from their partners, resulting in a better understanding of PNC, leading to good PNC perceptions. According to the present study, women who delivered their most recent child *via* caesarean section had a higher likelihood of having positive PNC perceptions. The finding might be the result of providing more care to women who would have delivered *via* caesarean section in anticipation of complications, which might produce favourable PNC experiences.

The present study found that women who did not attend ANC and PNC visits after the delivery of their last child were less likely to have positive PNC perceptions. These findings align with those of a study conducted in Ethiopia.^29^ This might be because of unaddressed misconceptions or negative beliefs about PNC if they lacked exposure to healthcare workers who could provide accurate information. Furthermore, ANC attendance provides women with information regarding potential complications of PNC and encourages them to utilise PNC services. This study did not find a statistically significant association between age and perceptions of PNC, although a positive correlation was initially expected. Older mothers often receive more focused attention during ANC and PNC visits because of perceived higher risk factors. This could be an interesting avenue for future research with a larger sample size or a more nuanced approach to capturing age-related risk factors. Another surprising finding was the absence of a statistically significant association between the place of delivery and perceptions of PNC. This might have been caused by the quality of care women receive from healthcare facilities. A study conducted in SSA revealed that disrespectful care was associated with negative PNC perceptions.^10^ Further research exploring the quality of care experiences in home and facility deliveries within this region could be insightful.

We recommend empowering women through education and income-generating projects to enhance their understanding of PNC information and to ensure they have the resources necessary to attend both ANC and PNC services, where such information is provided. Furthermore, the use of community health workers (CHWs) in disseminating PNC information to women is recommended so that even those who do not attend maternal healthcare services are informed about the importance of PNC. In addition, an improvement in the provision of information about contraception so that women in the region can limit their family sizes is recommended, since women with high parity are less likely to have good PNC knowledge. To improve PNC perceptions, it is recommended that the region devise strategies to increase both ANC and PNC attendance. Furthermore, the ANC and PNC services should be of good quality to ensure positive experiences, resulting in positive PNC experiences.

Recall bias might have impacted the study’s findings. This is because some participants had delivered 5 years before this study. However, the findings are considered generalisable to the rest of the region, since all public healthcare facilities were included in the study. The findings of this study may also apply to other regions of the country with the same population characteristics as the Oshana region.

## Conclusion

The study revealed that, although 56% of the participants had good PNC knowledge, only 27% had good PNC perceptions. Participants’ education levels, employment statuses, parity and attendance at ANC and PNC services all impacted their PNC knowledge. Marital status, the method of delivery of the previous child, awareness of PNC services and attendance at ANC and PNC services all had an impact on participants’ perceptions of PNC. Therefore, the empowerment of women through education and income-generating projects and also the use of CHWs to improve PNC knowledge in the Oshana region are recommended. An improvement in the provision of information on contraception to promote smaller family sizes is also recommended. Strategies should also be developed to enhance the utilisation of good-quality ANC and PNC services to improve PNC perceptions among women in the region. Furthermore, it is essential that both ANC and PNC services maintain good qualities in them to ensure women have positive experiences.
